# Lessons from Human Islet Transplantation Inform Stem Cell-Based Approaches in the Treatment of Diabetes

**DOI:** 10.3389/fendo.2021.636824

**Published:** 2021-03-11

**Authors:** Taylor M. Triolo, Melena D. Bellin

**Affiliations:** ^1^ The Barbara Davis Center for Diabetes, School of Medicine, University of Colorado Denver, Aurora, CO, United States; ^2^ Department of Pediatrics, School of Medicine, University of Minnesota, Minneapolis, MN, United States

**Keywords:** islet transplantation, type 1 diabetes, diabetes treatment, total pancreatectomy with islet autotransplant (TPIAT), allotransplantation, type 2 diabetes, beta cell transplantation, stem cell differentiation

## Abstract

Diabetes mellitus is characterized by the body’s inability to control blood glucose levels within a physiological range due to loss and/or dysfunction of insulin producing beta cells. Progressive beta cell loss leads to hyperglycemia and if untreated can lead to severe complications and/or death. Treatments at this time are limited to pharmacologic therapies, including exogenous insulin or oral/injectable agents that improve insulin sensitivity or augment endogenous insulin secretion. Cell transplantation can restore physiologic endogenous insulin production and minimize hyper- and hypoglycemic excursions. Islet isolation procedures and management of transplant recipients have advanced over the last several decades; both tight glycemic control and insulin independence are achievable. Research has been conducted in isolating islets, monitoring islet function, and mitigating the immune response. However, this procedure is still only performed in a small minority of patients. One major barrier is the scarcity of human pancreatic islet donors, variation in donor pancreas quality, and variability in islet isolation success. Advances have been made in generation of glucose responsive human stem cell derived beta cells (sBCs) and islets from human pluripotent stem cells using directed differentiation. This is an emerging promising treatment for patients with diabetes because they could potentially serve as an unlimited source of functional, glucose-responsive beta cells. Challenges exist in their generation including long term survival of grafts, safety of transplantation, and protection from the immune response. This review focuses on the progress made in islet allo- and auto transplantation and how these advances may be extrapolated to the sBC context.

## Introduction

### Patients With Insulin Deficient Forms of Diabetes May Be Considered for Islet Transplantation

Diabetes is characterized by the body’s inability to control blood glucose within a tight physiological range, due to insulin deficiency from beta cell loss or dysfunction and/or insulin resistance. Prolonged or recurrent hyperglycemia can lead to macro and microvascular complications, associated with substantial morbidity and early mortality (1, 2).

Type 1 diabetes is an autoimmune attack on a patient’s own insulin producing beta cells. If left untreated, severe insulin deficiency leads to hyperglycemia, diabetic ketoacidosis and potentially death. Treatment options at this time are limited to exogenous insulin; although progress has been made in the precision of delivery of insulin and blood glucose monitoring (2), patients are at risk for life-threatening hypoglycemia (3). While therapies targeted specifically at risk relatives have shown some promise in delaying onset of disease (4) there is still no cure for type 1 diabetes. Allogeneic islet transplantation may be considered in highly selected type 1 diabetes patients with either repeated severe hypoglycemic, significant glycemic variability, or microvascular complications (usually renal failure necessitating kidney transplant) (5). In the United States islet allotransplant is considered investigational and only performed in the context of a research study but is offered as standard clinical care in areas of Canada, Europe, and elsewhere. However, only a limited supply of suitable cadaveric donor pancreases are available for islet isolation and transplant.

In contrast, type 2 diabetes is a condition of insulin resistance and progressive beta cell decline (6). Patients are often treated with oral or injectable agents that improve endogenous insulin function. In some cases, patients with type 2 diabetes are also treated with insulin. Because the large islet mass needed to overcome insulin resistance is unlikely to be obtained with isolated islets, patients with type 2 diabetes are generally not considered candidates for islet transplantation. However, a renewable cell source could overcome this barrier of insufficient islet mass.

While type 1 and 2 diabetes are the more common causes of glucose dysfunction, beta cell loss and dysfunction can also occur in the setting of persistent inflammation and stress within the pancreas due to chronic pancreatitis (7). This is a painful and disabling condition that can be treated with analgesics, procedural interventions, and sometimes removal of the pancreas, rendering the patient without endocrine or exocrine pancreatic function. Total pancreatectomy and intraportal islet cell autotransplantation (TPIAT) can provide pain relief and sustained islet graft function in these patients. In this procedure, patients receive their own islets and therefore donor pancreases are not required. However, because of the damage and fibrosis from pancreatitis, these individuals usually have a sub-optimal islet mass and only about 1 out of every 3 achieves insulin independence.

In these etiologies of dysglycemia, replacement of beta cell function is a potential treatment to alleviate glycemic variability and reduce risk for the complications associated with long term hyperglycemia. A common challenge is obtaining a sufficient number of islets to successfully treat individual patients and offer cell therapy to a larger number of patients with diabetes. Here we review the uses of allo- and auto- islet transplantation and how stem cell derived beta cells (sBCs) or islets may overcome barriers and limitations currently inherent in islet transplantation (**Table 1**).

**Table 1:** Considerations in islet transplantation.

	Allotransplantation	Autotransplantation	Stem cell transplantation
Patient Population	Patients with type 1 diabetes and severe hypoglycemia, glycemic variability or microvascular complications	Patients with chronic pancreatitis undergoing pancreatectomy	Investigational; type 1 diabetes with potential application to other forms of diabetes
Source of Islet Material	Cadaveric islets	Autologous transplant	Human pluripotent stem cells (induced pluripotent stem cells or embryonic stem cells)
Limitations	- Immunosuppression required - Limited supply of donors	- Limited cell mass, from one’s own diseased pancreas	-Remains investigational - Need to scale up to sufficient functional mass to reverse diabetes in humans

### Islet Allotransplantation

Allogenic transplantation of cadaveric islets as a functional source of beta cells has become a treatment for patients with type 1 diabetes, particularly those with either life-threatening hypoglycemia or diabetes-related kidney failure requiring kidney transplantation. Although whole organ pancreas transplants can also be performed for these individuals (8–10), the appeal of islet transplantation is the lack of major surgery and very low risk for procedural complications. There have been improvements in isolation of islets and the procedure is considered minimally invasive. Initial transplants of pancreatic islets were trialed as early as the 1970s, with initially low rates of success (11). A turning point came with the introduction of the Edmonton Protocol in 2000 (12)—by introducing glucocorticoid-free immunosuppression and using multiple donors to increase islet mass, all 7 patients transplanted in the initial Edmonton trial achieved insulin independence. Subsequent refinements in immunosuppression protocols have improved the longevity of insulin independence (5, 13).

Even when insulin independence is not achieved, islet transplantation is highly successful in preventing severe hypoglycemia, if islet graft function is maintained (5, 14, 15). While success of islet transplants have generally improved over the last 20 years (5), variability in achieving insulin independence and concern for immunosuppression impact on kidney function (16) remain a concern. In addition, donor tissue availability continues to be the limiting factor of allogenic islet transplantation as a treatment for diabetes as 2–3 donors are typically required to obtain the necessary beta cell mass required for transplantation (17).

Despite improving long-term outcomes after islet allotransplantation, challenges remain around the longevity of insulin independence. Transplanted islets are subjected to non-immune attrition, and at risk for alloimmune rejection and recurrent autoimmunity (18–20). Immunosuppressive drugs necessary for islet allotransplant also carry risk for beta cell toxicity (21). Although intraportally transplanted islets are rarely accessible for study, limited histopathology of intraportal islet allografts have shown amyloid deposition, postulated due to over-stimulation of insulin production from a marginal islet mass or immunosuppressive drug toxicity (22); a recent report documenting absence of islet amyloid in an islet autograft patient with marginal islet mass suggests drug toxicity as a more likely culprit (23). More recently de-differentiation of the mature beta cell phenotype was observed in two islet allotransplant recipients, possibly consequences of hypoxia and metabolic stress (23). Innate immune destruction of islets stimulated upon intraportal infusion of islets has led to study of alternate sites for transplant, including omentum, bone marrow, intramuscular, and subcutaneous sites, though none has yet established the same efficacy as the liver (24–27).

As with any organ transplant, alloimmune rejection can occur in islet transplant and is more common in patients exhibiting high levels of HLA-sensitization pre-transplant (28–31). Unfortunately, immune rejection is difficult to treat due to limitations in early detection and lack of effective treatment strategies (10, 18, 32–34). Genetically engineered human beta cell lines can be used *in vivo* to augment the immune response to evaluate immune interactions and perhaps protect transplanted beta cells from immune destruction (35). Recurrent autoimmunity has been associated with positive autoantibodies, but the presence of autoreactive T-cell studies is more strongly associated with islet graft failure (36–39). Potential strategies to address these immunologic losses include encapsulation and use of bioengineered scaffold devices with enhanced vascularization and/or local drug release.

### Islet Autotransplantation

TPIAT is a treatment option for patients suffering from intractable abdominal pain from chronic pancreatitis. Total pancreatectomy provides pain relief by removing the primary source of chronic pain but results in complete exocrine insufficiency and insulin deficient diabetes. By combining total pancreatectomy with islet transplantation, patients can maintain some beta cell mass with insulin secretory capacity, in order to mitigate the severity of post-operative diabetes (40). Unlike allotransplantation, TPIAT does not require immunosuppression and patients serve as their own islet donors (41). Rather the challenge with islet transplantation is obtaining a sufficient number or mass of islets from a diseased pancreas.

TPIAT was first performed in the 1970s at the University of Minnesota (42) and since then has been adapted to many centers worldwide as a treatment for chronic pancreatitis (43–46). Because of the limitations in obtaining sufficient islet mass, only around one out of every three individuals is insulin independent after the procedure, but the majority preserve some endogenous insulin secretion benefiting glycemic control (41). Younger age and higher islet mass transplantation are predictors for functional graft survival (47) and normal preoperative glucose status can also improve post-operative graft success (48). Improvements have been made in the isolation of the islets and minimization of ischemia to the pancreatic islets (41). Work has been done to minimize risk of ischemia by avoiding prolonged cold ischemia to the isolated tissue but length of time has not been shown to have a detrimental effect on islet isolation and location of isolation (remote or onsite) does not affect insulin independence (49). Although some patients have maintained insulin independence for >10 years after TPIAT, as seen with Considerations in islet transplantation, insulin independence, and islet graft function wane over time (50). It is possible that metabolic strain due to glucotoxicity, exposure to toxins and medications (51) or the inability for islet neogenesis to occur in the liver (52) may contribute to the observed decline in islet graft function. An autologous renewable cell source could address the diabetes challenges after TPIAT by increasing islet mass and providing potential to “redose” islets later to address the apparent slow loss of islet mass over time after the procedure. For obvious reasons, autologous transplantation can only occur once in a patient’s lifetime and cadaveric islets require a deceased donor, therefore a source of renewable islet sources could benefit patients with islet dysfunction, either due to diabetes or chronic pancreatitis.

### Moving Beyond Allo and Autotransplantation

There is clearly a need for access to a renewable cell source for allotransplantation, and for re-transplant after autotransplantation or in chronic pancreatitis. There are some key lessons to be learned from allo- and autotransplantation for the future of cell therapy. From islet autotransplantation, we have functional data to establish a dose-response in the absence of targeted immunity– Islet graft function (C-peptide positivity) is nearly universal when a minimum threshold of 5,000 IEQ/kg is transplanted in the autograft setting, suggesting this may be an appropriate minimum “dose” target for a stem cell-derived therapy [particularly if immune barriers are fully addressed (41)]. Islet attrition occurs due to immune and non-immune stressors, and thus engineering the proper microenviroment for renewable cell sources may enhance the potential for long-term benefit. Encapsulation, engineered scaffolds, and alternate transplant sites are particularly relevant to stem cell therapy, where encapsulation may also both immunoprotect and to “contain” the cell product and sites outside the liver may be desired for safety. Addressing auto and alloimmunity, such as through encapsulation approaches, will continue to be a need for stem cell derived therapy.

### A Future for Stem Cell Derived Islets

**Figure 1:**
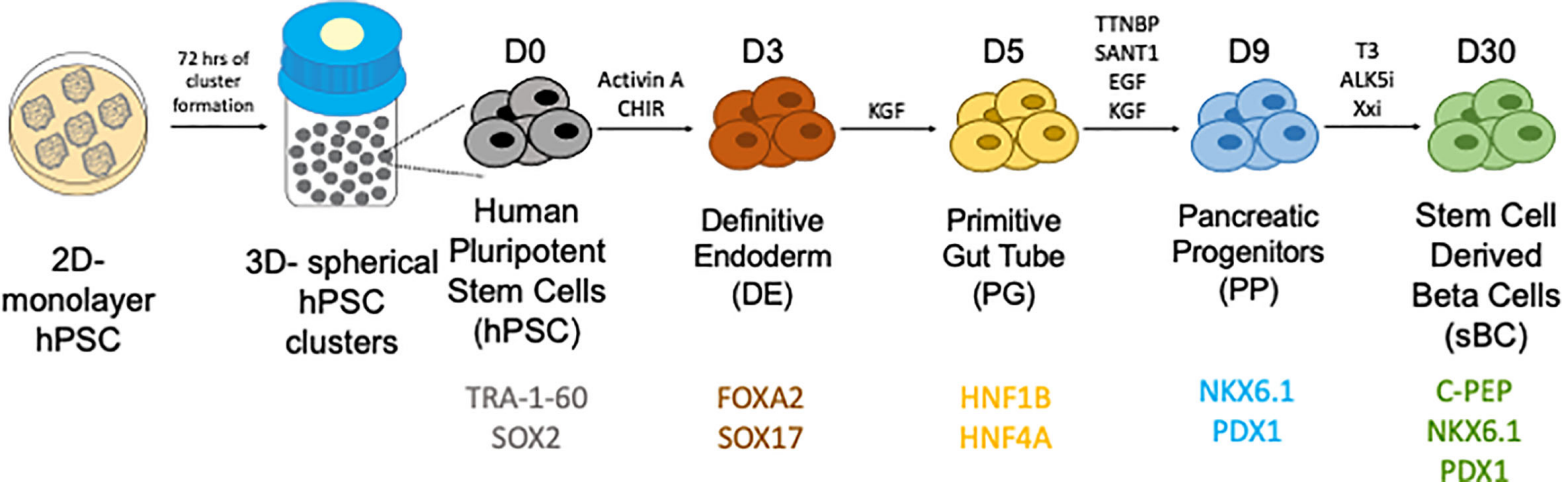
Generation of stem cell derived beta cells from human pluripotent stem cells through a stepwise differentiation protocol. Courtesy of Dr. Holger Russ.

Given the limitations of donor availability, the generation of glucose responsive human sBCs and islets from human pluripotent stem cells (hPSCs) are a potential future treatment for those with diabetes. Both human embryonic stem cells (hESCs) and induced pluripotent stem cells (iPSCs) are potential sources of hPSCs from which sBCs can be generated. hESCs are have been derived from blastocysts (53, 54). iPSCs are somatic cells that can be taken from a patient blood sample or fibroblast and reprogrammed with defined factors to the pluripotent state (55). Both hESCs and iPSCs are able to undergo differentiation and self-renewal to generate an unlimited source of potentially therapeutic cells. Much work has been done to direct the differentiation from hPSCs to the pancreatic lineage through stepwise differentiation protocols (56–59) (**Figure 1**). These cells are functionally mature (60) and display insulin secretory properties similar to human islets (61–63). Transplantation of stem cell derived pancreatic endoderm can mature to functional islets *in vivo* in rodents (58). Further work has been done to make this process functional and scalable (64). While in their infancy, current and future studies are underway in humans to investigate safety and efficacy of hPSC derived islets (NCT02239354, NCT03163511, and NCT02939118). These advances are the initial steps to providing renewable, functional islets to patients with beta cell dysfunction (65).

While great progress has been made in the development of these sBCs, there are several challenges and factors to consider. One factor to consider is the presence of off-target or undifferentiated cells that could interfere with the functional sBCs or be tumorigenic. Current and future clinical approaches using partially mature (pancreatic endoderm) or fully mature islets for implantation may reduce this risk, but close clinical follow up will be needed.

A second consideration is the role of immunogenicity of the sBCs or stem-cell derived islets. hESC derived cells, in the absence of genome editing, are subject to risk for alloimmune rejection which may require immunosuppression or encapsulation. Using iPSCs subverts this risk of alloimmunity and would be ideal for a cell source in TPIAT. Autologous sBCs have been successfully derived from patients with type 1 diabetes (62), but in this setting would remain at risk for autoimmune attack by autoreactive T-cells against pancreatic beta cells characteristic of type 1 diabetes. Therapeutic strategies for delaying this autoimmune attack have shown promise in at-risk relatives (4) but there is not yet an accepted therapy for halting this immune response. Even in the setting of allogeneic islet or simultaneous pancreas-kidney transplantation, where multi-drug immunosuppression is administered, islet autoimmunity can recur (66). Transplanted islet exosome profiling can be used as a way to monitor for evidence of recurrent autoimmunity (67). This can be tracked from a peripheral blood sample from a patient and may be a marker of beta cell injury patients who have undergone islet transplantation. Plasma detection of glutamate decarboxylase (GAD-65) can serve as a marker of beta cell loss after transplantation (68). Although hPSCs can evade allogenic response (69) once fully differentiated, these cells lose their immunologic privilege (70). Additionally, strategies being explored include the use of genetically engineered immune silent cells. Advances in genome engineering using CRISPR/Cas9 allows for modification of hPSCs (71) and can knock out HLA surface molecules implicated in autoimmunity (72, 73).

Pluripotent stem cells may be better poised to overcome the immunologic challenges of allotransplantation when combined with genetic engineering, encapsulation, or scaffolding technology. Bioengineered scaffolds offer novel opportunity to improve islet vascularization and optimize the islet microenvironment to protect grafts (27, 74, 75). Macroencapsulation of sBCs have been explored as a way to protect sBCs *in vivo* (76, 77) which could block transplanted sBCs from an immune attack but provide an environment to allow the survival of transplanted tissue. Trials are underway of to encapsulate sBCs for transplantation (78). These considerations will be important aspects to consider prior to considering transplantation of sBCs.

## Discussion

While much progress has been made in transplantation functional islet tissue as a treatment for diabetes, there are still many aspects that must be faced. While sBCs and islets can be generated on a large scale, there are still challenges to ensure protection from rejection, continued functionality and assurance of safety. Lessons learned from allo and auto islet transplantation will be helpful to apply in the sBC context.

## Author Contributions

TT is the guarantor of this work and, as such, takes responsibility for the integrity of the data and the accuracy of the data analysis. TT researched data and wrote the manuscript. MB researched data and reviewed/edited manuscript. All authors contributed to the article and approved the submitted version.

## Funding

TT is funded by the NIDDK K12 training grant (K12DK094712). The contents of this Article are solely the responsibility of the authors and do not necessarily represent the official views of the NIH.

## Conflict of Interest

MB discloses the following potential conflicts: Research grant support from Viacyte and Dexcom. Medical advisory role (DSMB) for Insulet.

The remaining author declares that the research was conducted in the absence of any commercial or financial relationships that could be construed as a potential conflict of interest.

## References

[1] Diabetes Control and Complications Trial Research Group Nathan DM Genuth S Lachin J Cleary P Crofford O. The effect of intensive treatment of diabetes on the development and progression of long-term complications in insulin-dependent diabetes mellitus. N Engl J Med (1993) 329(14):977–86. 10.1056/NEJM199309303291401 8366922

[2] Diabetes Control and Complications Trial (DCCT)/Epidemiology of Diabetes Interventions and Complications (EDIC) Research Group Lachin JM White NH Hainsworth DP Sun W Cleary PA. Effect of intensive diabetes therapy on the progression of diabetic retinopathy in patients with type 1 diabetes: 18 years of follow-up in the DCCT/EDIC. Diabetes (2015) 64(2):631–42. 10.2337/db14-0930 PMC4303965 25204977

[3] Cryer PE. Mechanisms of hypoglycemia-associated autonomic failure in diabetes. N Engl J Med (2013) 369(4):362–72. 10.1056/NEJMra1215228 23883381

[4] An Anti-CD3 Antibody, Teplizumab, in Relatives at Risk for Type 1 Diabetes. N Engl J Med (2020) 382(6):586. 10.1056/NEJMx190033 32023396

[5] Barton FB Rickels MR Alejandro R Hering BJ Wease S Naziruddin B. Improvement in outcomes of clinical islet transplantation: 1999-2010. Diabetes Care (2012) 35(7):1436–45. 10.2337/dc12-0063 PMC3379615 22723582

[6] Ferrannini E. Insulin resistance versus insulin deficiency in non-insulin-dependent diabetes mellitus: problems and prospects. Endocr Rev (1998) 19(4):477–90. 10.1210/edrv.19.4.0336 9715376

[7] Sasikala M Talukdar R Pavan kumar P Radhika G Rao GV Pradeep R. beta-Cell dysfunction in chronic pancreatitis. Dig Dis Sci (2012) 57(7):1764–72. 10.1007/s10620-012-2086-7 22383081

[8] Bosi E Bottazzo GF Secchi A Pozza G Shattock M Saunders A. Islet cell autoimmunity in type I diabetic patients after HLA-mismatched pancreas transplantation. Diabetes (1989) 38(Suppl 1):82–4. 10.2337/diab.38.1.S82 2642861

[9] Braghi S Bonifacio E Secchi A Di Carlo V Pozza G Bosi E. Modulation of humoral islet autoimmunity by pancreas allotransplantation influences allograft outcome in patients with type 1 diabetes. Diabetes (2000) 49(2):218–24. 10.2337/diabetes.49.2.218 10868938

[10] Vendrame F Pileggi A Laughlin E Allende G Martin-Pagola A Molano RD. Recurrence of type 1 diabetes after simultaneous pancreas-kidney transplantation, despite immunosuppression, is associated with autoantibodies and pathogenic autoreactive CD4 T-cells. Diabetes (2010) 59(4):947–57. 10.2337/db09-0498 PMC2844842 20086230

[11] Scharp DW Lacy PE Santiago JV McCullough CS Weide LG Falqui L. Insulin independence after islet transplantation into type I diabetic patient. Diabetes (1990) 39(4):515–8. 10.2337/diabetes.39.4.515 2108071

[12] Shapiro AM Lakey JR Ryan EA Korbutt GS Toth E Warnock GL. Islet transplantation in seven patients with type 1 diabetes mellitus using a glucocorticoid-free immunosuppressive regimen. N Engl J Med (2000) 343(4):230–8. 10.1056/NEJM200007273430401 10911004

[13] Bellin MD Barton FB Heitman A Harmon JV Kandaswamy R Balamurugan AN. Potent induction immunotherapy promotes long-term insulin independence after islet transplantation in type 1 diabetes. Am J Transplant (2012) 12(6):1576–83. 10.1111/j.1600-6143.2011.03977.x PMC3390261 22494609

[14] Hering BJ Clarke WR Bridges ND Eggerman TL Alejandro R Bellin MD. Phase 3 Trial of Transplantation of Human Islets in Type 1 Diabetes Complicated by Severe Hypoglycemia. Diabetes Care (2016) 39(7):1230–40. 10.2337/dc15-1988 PMC5317236 27208344

[15] Ryan EA Shandro T Green K Paty BW Senior PA Bigam D. Assessment of the Severity of Hypoglycemia and Glycemic Lability in Type 1 Diabetic Subjects Undergoing Islet Transplantation. Diabetes (2004) 53(4):955–62. 10.2337/diabetes.53.4.955 15047610

[16] Senior PA Zeman M Paty BW Ryan EA Shapiro AM. Changes in renal function after clinical islet transplantation: four-year observational study. Am J Transplant (2007) 7(1):91–8. 10.1111/j.1600-6143.2006.01573.x 17227560

[17] Ryan EA Lakey JRT Rajotte RV Korbutt GS Kin T Imes S. Clinical Outcomes and Insulin Secretion After Islet Transplantation With the Edmonton Protocol. Diabetes (2001) 50(4):710–9. 10.2337/diabetes.50.4.710 11289033

[18] Huurman VA Hilbrands R Pinkse GG Gillard P Duinkerken G van de Linde P. Cellular islet autoimmunity associates with clinical outcome of islet cell transplantation. PloS One (2008) 3(6):e2435. 10.1371/journal.pone.0002435 18560516 PMC2426735

[19] Roelen DL Huurman VA Hilbrands R Gillard P Duinkerken G van der Meer-Prins PW. Relevance of cytotoxic alloreactivity under different immunosuppressive regimens in clinical islet cell transplantation. Clin Exp Immunol (2009) 156(1):141–8. 10.1111/j.1365-2249.2008.03812.x PMC2673752 19161445

[20] Qi M Kinzer K Danielson KK Martellotto J Barbaro B Wang Y. Five-year follow-up of patients with type 1 diabetes transplanted with allogeneic islets: the UIC experience. Acta Diabetol (2014) 51(5):833–43. 10.1007/s00592-014-0627-6 PMC4801517 25034311

[21] Triñanes J Rodriguez-Rodriguez AE Brito-Casillas Y Wagner A De Vries APJ Cuesto G. Deciphering Tacrolimus-Induced Toxicity in Pancreatic β Cells. Am J Transplant (2017) 17(11):2829–40. 10.1111/ajt.14323 28432716

[22] Westermark GT Westermark P Berne C Korsgren O. Widespread amyloid deposition in transplanted human pancreatic islets. N Engl J Med (2008) 359(9):977–9. 10.1056/NEJMc0802893 18753660

[23] Generette GS Bachul PJ Boylan KE Yassan LJ Hart J Pyda JS. Neither amyloid depositions nor hepatic steatosis are associated with marginal islet mass early after autotransplantation. Am J Transplant (2020) 0:1–3. 10.1111/ajt.16406 33217154

[24] Maffi P Nano R Monti P Melzi R Sordi V Mercalli A. Islet Allotransplantation in the Bone Marrow of Patients With Type 1 Diabetes: A Pilot Randomized Trial. Transplantation (2019) 103(4):839–51. 10.1097/TP.0000000000002416 30130323

[25] Bertuzzi F Colussi G Lauterio A De Carlis L. Intramuscular islet allotransplantation in type 1 diabetes mellitus. Eur Rev Med Pharmacol Sci (2018) 22(6):1731–6. 10.26355/eurrev_201803_14588 29630119

[26] Baidal DA Ricordi C Berman DM Alvarez A Padilla N Ciancio G. Bioengineering of an Intraabdominal Endocrine Pancreas. N Engl J Med (2017) 376(19):1887–9. 10.1056/NEJMc1613959 PMC5572072 28489987

[27] Carlsson PO Espes D Sedigh A Rotem A Zimerman B Grinberg H. Transplantation of macroencapsulated human islets within the bioartificial pancreas βAir to patients with type 1 diabetes mellitus. Am J Transplant (2018) 18(7):1735–44. 10.1111/ajt.14642 PMC6055594 29288549

[28] Campbell PM Salam A Ryan EA Senior P Paty BW Bigam D. Pretransplant HLA antibodies are associated with reduced graft survival after clinical islet transplantation. Am J Transplant (2007) 7(5):1242–8. 10.1111/j.1600-6143.2007.01777.x 17456201

[29] Piemonti L Everly MJ Maffi P Scavini M Poli F Nano R. Alloantibody and autoantibody monitoring predicts islet transplantation outcome in human type 1 diabetes. Diabetes (2013) 62(5):1656–64. 10.2337/db12-1258 PMC3636624 23274902

[30] Mohanakumar T Narayanan K Desai N Ramachandran S Shenoy S Jendrisak M. A significant role for histocompatibility in human islet transplantation. Transplantation (2006) 82(2):180–7. 10.1097/01.tp.0000226161.82581.b2 16858280

[31] Olack BJ Swanson CJ Flavin KS Phelan D Brennan DC White NH. Sensitization to HLA antigens in islet recipients with failing transplants. Transplant Proc (1997) 29(4):2268–9. 10.1016/S0041-1345(97)00327-8 9193621

[32] Hilbrands R Huurman VA Gillard P Velthuis JH De Waele M Mathieu C. Differences in baseline lymphocyte counts and autoreactivity are associated with differences in outcome of islet cell transplantation in type 1 diabetic patients. Diabetes (2009) 58(10):2267–76. 10.2337/db09-0160 PMC2750206 19602536

[33] Laughlin E Burke G Pugliese A Falk B Nepom G. Recurrence of autoreactive antigen-specific CD4+ T cells in autoimmune diabetes after pancreas transplantation. Clin Immunol (2008) 128(1):23–30. 10.1016/j.clim.2008.03.459 18455963 PMC2531116

[34] Monti P Scirpoli M Maffi P Ghidoli N De Taddeo F Bertuzzi F. Islet transplantation in patients with autoimmune diabetes induces homeostatic cytokines that expand autoreactive memory T cells. J Clin Invest (2008) 118(5):1806–14. 10.1172/JCI35197 PMC2323193 18431516

[35] van der Torren CR Zaldumbide A Roelen DL Duinkerken G Brand-Schaaf SH Peakman M. Innate and adaptive immunity to human beta cell lines: implications for beta cell therapy. Diabetologia (2016) 59(1):170–5. 10.1007/s00125-015-3779-1 PMC4670455 26489735

[36] Pinkse GG Tysma OH Bergen CA Kester MG Ossendorp F van Veelen PA. Autoreactive CD8 T cells associated with beta cell destruction in type 1 diabetes. Proc Natl Acad Sci U S A (2005) 102(51):18425–30. 10.1073/pnas.0508621102 PMC1317949 16339897

[37] Unger WW Velthuis J Abreu JR Laban S Quinten E Kester MG. Discovery of low-affinity preproinsulin epitopes and detection of autoreactive CD8 T-cells using combinatorial MHC multimers. J Autoimmun (2011) 37(3):151–9. 10.1016/j.jaut.2011.05.012 21636247

[38] Velthuis JH Unger WW van der Slik AR Duinkerken G Engelse M Schaapherder AF. Accumulation of autoreactive effector T cells and allo-specific regulatory T cells in the pancreas allograft of a type 1 diabetic recipient. Diabetologia (2009) 52(3):494–503. 10.1007/s00125-008-1237-z 19104770

[39] Velthuis JH Unger WW Abreu JR Duinkerken G Franken K Peakman M. Simultaneous detection of circulating autoreactive CD8+ T-cells specific for different islet cell-associated epitopes using combinatorial MHC multimers. Diabetes (2010) 59(7):1721–30. 10.2337/db09-1486 PMC2889772 20357361

[40] McEachron KR Bellin MD. Total pancreatectomy and islet autotransplantion for chronic and recurrent acute pancreatitis. Curr Opin Gastroenterol (2018) 34(5):367–73. 10.1097/MOG.0000000000000458 PMC9623823 29901515

[41] Sutherland DE Radosevich DM Bellin MD Hering BJ Beilman GJ Dunn TB. Total pancreatectomy and islet autotransplantation for chronic pancreatitis. J Am Coll Surg (2012) 214(4):409–24; discussion 24-6. 10.1016/j.jamcollsurg.2011.12.040 22397977 PMC3755128

[42] Najarian JS Sutherland DE Matas AJ Goetz FC. Human islet autotransplantation following pancreatectomy. Transplant Proc (1979) 11(1):336–40. 109963

[43] Morgan KA Lancaster WP Owczarski SM Wang H Borckardt J Adams DB. Patient Selection for Total Pancreatectomy with Islet Autotransplantation in the Surgical Management of Chronic Pancreatitis. J Am Coll Surg (2018) 226(4):446–51. 10.1016/j.jamcollsurg.2017.12.018 29289751

[44] Wilson GC Sutton JM Abbott DE Smith MT Lowy AM Matthews JB. Long-Term Outcomes After Total Pancreatectomy and Islet Cell Autotransplantation: Is It a Durable Operation? Ann Surg (2014) 260(4):659–67. 10.1097/SLA.0000000000000920 25203883

[45] Garcea G Weaver J Phillips J Pollard CA Ilouz SC Webb MA. Total pancreatectomy with and without islet cell transplantation for chronic pancreatitis: a series of 85 consecutive patients. Pancreas (2009) 38(1):1–7. 10.1097/MPA.0b013e3181825c00 18665009

[46] Takita M Lara LF Naziruddin B Shahbazov R Lawrence MC Kim PT. Effect of the Duration of Chronic Pancreatitis on Pancreas Islet Yield and Metabolic Outcome Following Islet Autotransplantation. J Gastrointest Surg (2015) 19(7):1236–46. 10.1007/s11605-015-2828-x 25933581

[47] Chinnakotla S Beilman GJ Dunn TB Bellin MD Freeman ML Radosevich DM. Factors Predicting Outcomes After a Total Pancreatectomy and Islet Autotransplantation Lessons Learned From Over 500 Cases. Ann Surg (2015) 262(4):610–22. 10.1097/SLA.0000000000001453 PMC5554067 26366540

[48] Quartuccio M Hall E Singh V Makary MA Hirose K Desai N. Glycemic Predictors of Insulin Independence After Total Pancreatectomy With Islet Autotransplantation. J Clin Endocrinol Metab (2017) 102(3):801–9. 10.1210/jc.2016-2952 PMC5460683 27870552

[49] Kesseli SJ Smith KD Jung MK Lin YK Walsh RM Hatipoglu B. Islet Cell Yield Following Remote Total Pancreatectomy With Islet Autotransplant is Independent of Cold Ischemia Time. Pancreas (2017) 46(3):380–4. 10.1097/MPA.0000000000000792 PMC5308539 28129232

[50] Bellin MD Beilman GJ Sutherland DER Ali H Petersen A Mongin S. How Durable Is Total Pancreatectomy and Intraportal Islet Cell Transplantation for Treatment of Chronic Pancreatitis? J Am Coll Surg (2019) 228(4):329–39. 10.1016/j.jamcollsurg.2018.12.019 PMC8579321 30630085

[51] Ngo A Sutherland DE Beilman GJ Bellin MD. Deterioration of glycemic control after corticosteroid administration in islet autotransplant recipients: a cautionary tale. Acta Diabetol (2014) 51(1):141–5. 10.1007/s00592-011-0315-8 PMC3461234 21822910

[52] Soltani SM O’Brien TD Loganathan G Bellin MD Anazawa T Tiwari M. Severely fibrotic pancreases from young patients with chronic pancreatitis: evidence for a ductal origin of islet neogenesis. Acta Diabetol (2013) 50(5):807–14. 10.1007/s00592-011-0306-9 PMC4124082 21773756

[53] Thomson JA Itskovitz-Eldor J Shapiro SS Waknitz MA Swiergiel JJ Marshall VS. Embryonic stem cell lines derived from human blastocysts. Science (1998) 282(5391):1145–7. 10.1126/science.282.5391.1145 9804556

[54] Cowan CA Klimanskaya I McMahon J Atienza J Witmyer J Zucker JP. Derivation of Embryonic Stem-Cell Lines from Human Blastocysts. N Engl J Med (2004) 350(13):1353–6. 10.1056/NEJMsr040330 14999088

[55] Takahashi K Yamanaka S. Induction of Pluripotent Stem Cells from Mouse Embryonic and Adult Fibroblast Cultures by Defined Factors. Cell (2006) 126(4):663–76. 10.1016/j.cell.2006.07.024 16904174

[56] D’Amour KA Agulnick AD Eliazer S Kelly OG Kroon E Baetge EE. Efficient differentiation of human embryonic stem cells to definitive endoderm. Nat Biotechnol (2005) 23(12):1534–41. 10.1038/nbt1163 16258519

[57] Kroon E Martinson LA Kadoya K Bang AG Kelly OG Eliazer S. Pancreatic endoderm derived from human embryonic stem cells generates glucose-responsive insulin-secreting cells in vivo. Nat Biotechnol (2008) 26(4):443–52. 10.1038/nbt1393 18288110

[58] D’Amour KA Bang AG Eliazer S Kelly OG Agulnick AD Smart NG. Production of pancreatic hormone–expressing endocrine cells from human embryonic stem cells. Nat Biotechnol (2006) 24(11):1392–401. 10.1038/nbt1259 17053790

[59] Pagliuca Felicia W Millman Jeffrey R Gürtler M Segel M Van Dervort A Ryu Jennifer H. Generation of Functional Human Pancreatic β Cells In Vitro. Cell (2014) 159(2):428–39. 10.1016/j.cell.2014.09.040 PMC4617632 25303535

[60] Velazco-Cruz L Song J Maxwell KG Goedegebuure MM Augsornworawat P Hogrebe NJ. Acquisition of Dynamic Function in Human Stem Cell-Derived β Cells. Stem Cell Rep (2019) 12(2):351–65. 10.1016/j.stemcr.2018.12.012 PMC6372986 30661993

[61] Robert T De Mesmaeker I Stangé GM Suenens KG Ling Z Kroon EJ. Functional Beta Cell Mass from Device-Encapsulated hESC-Derived Pancreatic Endoderm Achieving Metabolic Control. Stem Cell Rep (2018) 10(3):739–50. 10.1016/j.stemcr.2018.01.040 PMC5918665 29503087

[62] Millman JR Xie C Van Dervort A Gürtler M Pagliuca FW Melton DA. Generation of stem cell-derived β-cells from patients with type 1 diabetes. Nat Commun (2016) 7:11463. 10.1038/ncomms11463 27163171 PMC4866045

[63] Nair GG Liu JS Russ HA Tran S Saxton MS Chen R. Recapitulating endocrine cell clustering in culture promotes maturation of human stem-cell-derived beta cells. Nat Cell Biol (2019) 21(2):263–74. 10.1038/s41556-018-0271-4 PMC6746427 30710150

[64] Schulz TC Young HY Agulnick AD Babin MJ Baetge EE Bang AG. A scalable system for production of functional pancreatic progenitors from human embryonic stem cells. PloS One (2012) 7(5):e37004. 10.1371/journal.pone.0037004 22623968 PMC3356395

[65] Henry RR Pettus J Wilensky J Shapiro AMJ Senior PA Roep B. Initial Clinical Evaluation of VC-01TM Combination Product—A Stem Cell–Derived Islet Replacement for Type 1 Diabetes (T1D). Diabetes (2018) 67(Supplement 1):138–OR. 10.2337/db18-138-OR

[66] Burke GW,3 Vendrame F Virdi SK Ciancio G Chen L Ruiz P. Lessons From Pancreas Transplantation in Type 1 Diabetes: Recurrence of Islet Autoimmunity. Curr Diabetes Rep (2015) 15(12):121. 10.1007/s11892-015-0691-5 26547222

[67] Korutla L Rickels MR Hu RW Freas A Reddy S Habertheuer A. Noninvasive diagnosis of recurrent autoimmune type 1 diabetes after islet cell transplantation. Am J Transplant (2019) 19(6):1852–8. 10.1111/ajt.15322 PMC7043773 30801971

[68] Pipeleers D Robert T De Mesmaeker I Ling Z. Concise Review: Markers for Assessing Human Stem Cell-Derived Implants as β-Cell Replacement in Type 1 Diabetes. Stem Cells Transl Med (2016) 5(10):1338–44. 10.5966/sctm.2015-0187 PMC5031173 27381993

[69] English K Wood KJ. Immunogenicity of embryonic stem cell-derived progenitors after transplantation. Curr Opin Organ Transplant (2011) 16(1):90–5. 10.1097/MOT.0b013e3283424faa 21150615

[70] van der Torren CR Zaldumbide A Duinkerken G Brand-Schaaf SH Peakman M Stangé G. Immunogenicity of human embryonic stem cell-derived beta cells. Diabetologia (2017) 60(1):126–33. 10.1007/s00125-016-4125-y PMC6518073 27787618

[71] Lin S Staahl BT Alla RK Doudna JA. Enhanced homology-directed human genome engineering by controlled timing of CRISPR/Cas9 delivery. Elife (2014) 3:e04766. 10.7554/eLife.04766 25497837 PMC4383097

[72] Wang D Quan Y Yan Q Morales JE Wetsel RA. Targeted Disruption of the β2-Microglobulin Gene Minimizes the Immunogenicity of Human Embryonic Stem Cells. Stem Cells Transl Med (2015) 4(10):1234–45. 10.5966/sctm.2015-0049 PMC4572902 26285657

[73] Han X Wang M Duan S Franco PJ Kenty JH Hedrick P. Generation of hypoimmunogenic human pluripotent stem cells. Proc Natl Acad Sci U S A (2019) 116(21):10441–6. 10.1073/pnas.1902566116 PMC6535035 31040209

[74] Nalbach L Roma LP Schmitt BM Becker V Körbel C Wrublewsky S. Improvement of islet transplantation by the fusion of islet cells with functional blood vessels. EMBO Mol Med (2020) 13(1):e12616. 10.15252/emmm.202012616 33135383 PMC7799357

[75] Elizondo DM Brandy NZD da Silva RLL de Moura TR Ali J Yang D. Pancreatic islets seeded in a novel bioscaffold forms an organoid to rescue insulin production and reverse hyperglycemia in models of type 1 diabetes. Sci Rep (2020) 10(1):4362. 10.1038/s41598-020-60947-x 32152396 PMC7062832

[76] Motte E Szepessy E Suenens K Stange G Bomans M Jacobs-Tulleneers-Thevissen D. Composition and function of macroencapsulated human embryonic stem cell-derived implants: comparison with clinical human islet cell grafts. Am J Physiol Endocrinol Metab (2014) 307(9):E838–46. 10.1152/ajpendo.00219.2014 25205822

[77] Haller C Piccand J De Franceschi F Ohi Y Bhoumik A Boss C. Macroencapsulated Human iPSC-Derived Pancreatic Progenitors Protect against STZ-Induced Hyperglycemia in Mice. Stem Cell Rep (2019) 12(4):787–800. 10.1016/j.stemcr.2019.02.002 PMC6449839 30853374

[78] Agulnick AD Ambruzs DM Moorman MA Bhoumik A Cesario RM Payne JK. Insulin-Producing Endocrine Cells Differentiated In Vitro From Human Embryonic Stem Cells Function in Macroencapsulation Devices In Vivo. Stem Cells Transl Med (2015) 4(10):1214–22. 10.5966/sctm.2015-0079 PMC4572906 26304037

